# In vivo study of light-driven naproxen release from gated mesoporous silica drug delivery system

**DOI:** 10.1038/s41598-021-99678-y

**Published:** 2021-10-12

**Authors:** Miroslav Almáši, Anna Alexovič Matiašová, Monika Šuleková, Eva Beňová, Juraj Ševc, Lucia Váhovská, Maksym Lisnichuk, Vladimír Girman, Adriana Zeleňáková, Alexander Hudák, Vladimír Zeleňák

**Affiliations:** 1 Department of Inorganic Chemistry, Institute of Chemistry, Faculty of Science, P.J. Šafárik University, Moyzesova 11, 041 54 Kosice, Slovakia; 2 Department of Cell Biology, Institute of Biology and Ecology, Faculty of Science, P. J. Šafárik University, Šrobárová 2, 041 54 Kosice, Slovakia; 3 Department of Chemistry, Biochemistry and Biophysics, Institute of Pharmaceutical Chemistry, The University of Veterinary Medicine and Pharmacy, Komenského 73, 041 81 Kosice, Slovakia; 4 Department of Condensed Matter Physics, Institute of Physics, Faculty of Science, P.J. Šafárik University, Park Angelinum 9, 041 54 Kosice, Slovakia

**Keywords:** Chemical biology, Drug discovery, Zoology, Chemistry, Materials science, Nanoscience and technology

## Abstract

A drug delivery system based on mesoporous particles MCM-41 was post-synthetically modified by photo-sensitive ligand, methyl-(2E)-3-(4-(triethoxysilyl)-propoxyphenyl)-2-propenoate (CA) and the pores of MCM-41 particles were loaded with Naproxen sodium salt (NAP). The CA was used as a photoactive molecule that can undergo a reversible photo-dimerization by [2π + 2π] cycloaddition when irradiated with UV light of specific wavelengths. Thus, it has a function of gate-keeper that is responsible for opening/closing the pores and minimizing premature release of NAP. The physicochemical properties of the prepared system were studied by infrared spectroscopy (IR), nitrogen adsorption measurements, thermogravimetric analysis (TGA), scanning transmission electron microscopy (STEM) and energy dispersive X-ray spectroscopy (EDX). The mechanism of the opening/closing pores was confirmed by UV measurements. In vitro and in vivo drug release experiments and the concentration of released NAP was determined by UV spectroscopy and high-performance liquid chromatography (HPLC). In vivo drug release in the blood circulatory system of rats has demonstrated the effective photo-cleavage reaction of CA molecules after UV-light stimulation. The localization and morphological changes of the particles were studied in the blood and liver of rats at different time intervals. The particles in the blood have been shown to retain their original rod-like shape, and the particles in the liver have been hydrolysed, which has resulted in spherical shape with a reduced size.

## Introduction

Development of nanotechnology and nanomedicine significantly influences the human everyday life and enables the knowledge gained in recent years to penetrate into the different fields, including medicine. Nanoparticles are used for diagnostic applications using imaging technology but also for treatment purposes through the drug delivery approach^1–4^. Nanoparticle based drug delivery have demonstrated enhanced efficacy and reduced side effects, due to the properties brought on by nanoscale^5–7^. Among different drug delivery systems (DDS), which revolutionized drug delivery studies in recent years, inorganic mesoporous silica nanoparticles (MSNs) show unique and superlative properties like excellent biocompatibility, high drug loading capacity, rigid framework, well-defined pore structure, easily controllable morphology, and tunable surface chemistry^5,8,9^. Their advantageous features make them ideal platforms to design multifunctional nanosystems. One of the major advantages of MSNs is the possibility to design zero-premature cargo release nanosystems using various gatekeepers and release the loaded drugs using different physical or chemical stimuli^10–14^. MSNs as delivery systems attracted a huge scientific interest and a number of interesting topical reviews are published annually by the leaders in the field, discussing the trends in research and application of MSNs and their use in modern medicine, with the visions that DDS technology based on MSNs might have in the near future^15–17^.

Due to interesting properties and great potential of MSNs as DDS, mesoporous silica materials are part of intensive research including in vivo studies. Brinker et al. used imaging-based pharmacokinetics to study in vivo porous silica nanoparticles. They observed monotonic decrease in systemic silica bioavailability with increasing particle size and corresponding accumulation in liver and spleen. The bioavailability was independed on the route of administration^18^. Bhavsar et al. studied in vitro and in vivo safety and degradation of MSNs^19^. They found, that MSNs are safe after *i.v.* administration up to 40 mg/kg and cause no acute or chronic toxicities. Moreover, it was found that MSNs are degradable and they are excreted from Wistar rats within 4 days after *i.v.* injection^19^. In other work, Nasr et al. used mesoporous silica nanoparticles for silymarin delivery^20^. The loading of silymarin on MSNs lead to an enhancement of silymarin bioavailability. The studied DDS showed no fatalities among rats within 22 days after oral administration and it was concluded that oral administration using MSNs based systems is a promising alternative for drug delivery with an accepted safety profile^20^. Mehmood et al. investigated MSNs loaded with drug velpatasvir using in vitro and in vivo experiments. The in vivo and in vitro studies showed on that loading of the drug to MSNs led to rapid drug absorption, high blood concentration and higher accumulation of the drug at the target site (liver), as compared to pure drug velpatasvir^21^.

**Figure 1:**
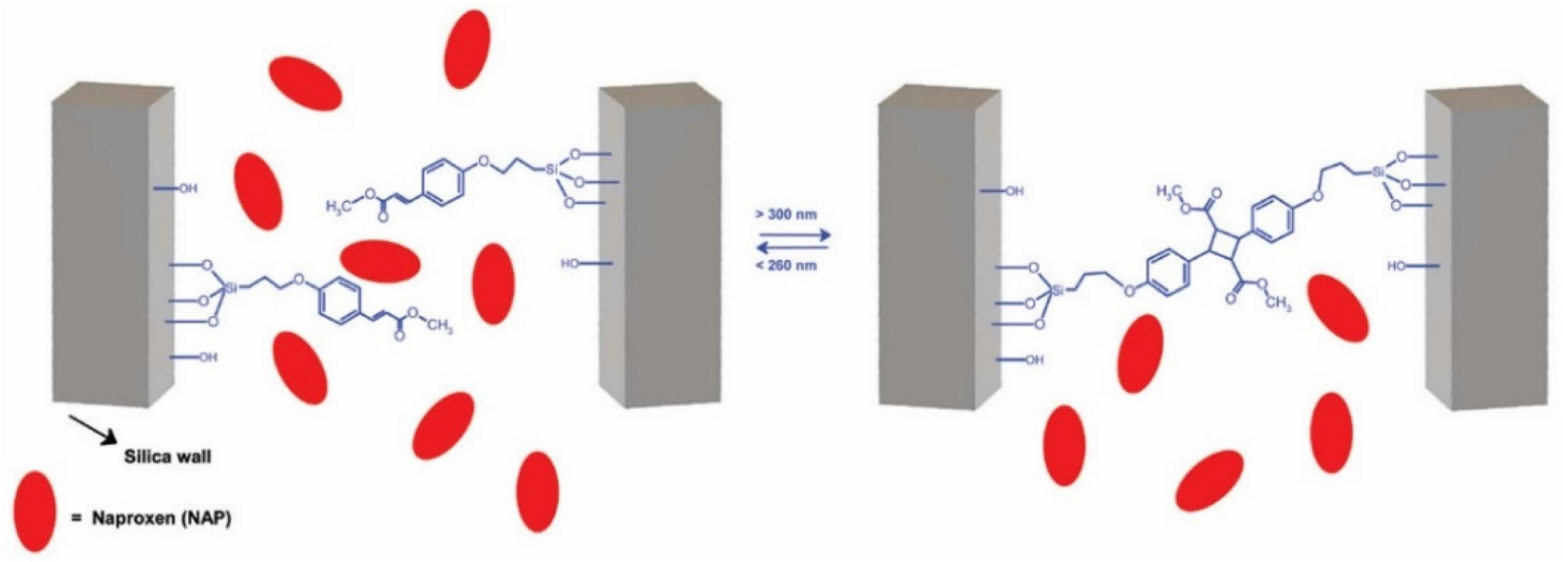
Mechanism of pore closing / pore opening using irradiation with UV light.

Based on this knowledge and great drug delivery potential of MSNs, in our previous works, we have described the different mesoporous silica particles for pH driven drug release^22,23^, redox potential driven release^24^, temperature driven drug release^25^ or light driven drug release^26,27^. In addition to physicochemical-characterisation, we studied biocompatibility and cytotoxicity of the prepared DDS in vitro on the U87 MG and SKBR3 cells using the microscopic techniques, flow-cytometry, MTT assay, apoptosis assay and CAM assay^22,23,28,29^. As a continuation of these works in the present study, we studied the functionality of such systems in vivo. For this purpose, we tested mesoporous photoactive silica nanoparticles loaded with the non-steroidal anti-inflammatory drug (NSAID) Naproxen sodium salt in model animals, Wistar rats. For the study, we have chosen the mesoporous silica DDS with light-driven drug release. The MSNs were modified with coumaric acid derivative, methyl-(2E)-3-(4-(triethoxysilyl)-propoxyphenyl)-2-propenoate (CA). The CA anchored on the surface of the silica is a biocompatible photo-sensitive organic compound that undergoes typical [2 + 2] dimerization upon irradiation with UV light (see Fig. 1).

## Methods and experiments

Chemicals used in the synthesis of MSNs and methyl-(2E)-3-(4-(triethoxysilyl)-propoxyphenyl)-2-propenoate were in the highest available quality and used without further purification. Solvents used in extraction and HPLC measurements were in HPLC grade. All chemicals were purchased from Sigma Aldrich, Acros Organics or Vibrac company.

### Synthesis of drug carrier

**Figure 2:**
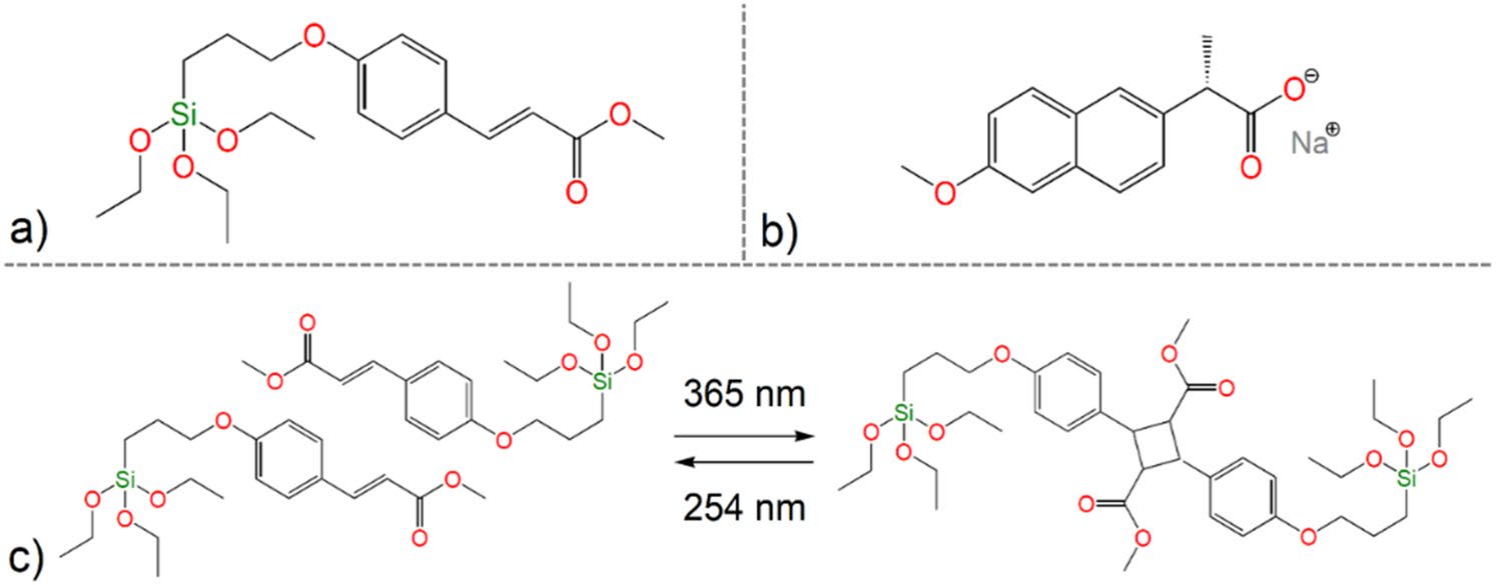
Molecular structure of (**a**) methyl-(2E)-3-(4-(triethoxysilyl)-propoxyphenyl)-2-propenoate (CA), (**b**) Naproxen sodium salt and (**c**) photo-dimerization/cleavage reaction of CA.

As a MSNs a mesoporous silica MCM-41 type was chosen. MCM-41 was prepared by sol–gel method with a molar ratio of reactants 1 TEOS (Tetraethyl orthosilicate, 98%, Sigma-Aldrich, CAS: 78-10-4, CN: 131903): 0.33 NaOH (≥ 98%, Sigma-Aldrich, CAS: 1310-73-2, CN: S5881): 0.12 CTAB (Hexadecyltrimethylammonium bromide, ≥ 98%, Sigma-Aldrich, CAS: 57–09-0, CN: H5882): 601.3 H_2_O^30^. NaOH solution was prepared by dissolution of 0.84 g sodium hydroxide in 730 g of water. Subsequently, 2.99 g of CTAB surfactant was added and the solution was stirred until the complete dissolution of CTAB. The reaction mixture was then treated dropwise with 14.00 g of tetraethoxysilane (TEOS) and stirred at laboratory temperature for 2 h. The as-synthesized product MCM-41 was washed several times with distilled water, filtered off and dried in the stream of air. After the drying procedure, surfactant molecules located in the pores of MCM-41 were removed by the calcination. MCM-41 was calcined in an oven with a slow heating rate of 0.5 °C. min^−1^ from laboratory temperature to 600 °C for 8 h. Photosensitive ligand methyl-(2E)-3-(4-(triethoxysilyl)-propoxyphenyl)-2-propenoate (CA, see Fig. 2a) was synthesized and grafted on the surface of prepared MCM-41 by the synthetic procedures described in^27^ and prepared material was denoted as MCM-41-CA.

### Drug loading

In general, drug loading was performed by the impregnation method in mass ratio 1 : 2 (MCM-41-CA: NAP). 300 mg of MCM-41-CA was dispersed in 5 mL of a drug solution consisting of 600 mg of Naproxen sodium salt (98%, Acros Organics, CAS: 26159-34-2, CN: 436710250, see Fig. 2b) and 5 mL of water. The suspensions were stirred at room temperature for 24 h while being irradiated with UV light, 254 nm to get an open pore configuration or 365 nm for closed pore configuration. Subsequently, the materials were separated, washed with a small amount of water, dried in a stream of air and stored in the dark. The prepared materials were denoted as MCM-41-CA/NAP (open) and MCM-41-CA/NAP (closed).

### Photo-dimerization reaction

Verification of the photo-dimerization reaction of the prepared cinnamic acid derivative (see Fig. 2c) was performed by irradiating (*λ* = 365 nm, 40 W) a methanol (anhydrous, 99.8%, Sigma Aldrich, CAS: 67-56-1, CN: 322415) solution of CA with a concentration of 5 × 10^–4^ M at time intervals of 5, 10 and 15 min. Dimerization reaction was monitored by UV spectroscopy.

### In vitro drug release experiment

5 mg of MCM-41-CA/NAP (closed) was dispersed in 10 cm^3^ of saline solution and stirred on a magnetic stirrer in the dark. The release amount of NAP was analysed at selected time intervals (0–16 min, every 2 min) using UV–VIS spectroscopy as follows: 2 mL of the solution at the specified time interval was taken into a 3 mL plastic vial and immediately centrifuged using an Eppendorf Minispin® microcentrifuge at 10 000 rpm for 30 s. 1 mL of supernatant was pipetted into a 3.5 mL glass cuvette with a 10 mm path length and the UV–VIS spectrum was measured in the wavelength range of 210–240 nm with a measurement speed of 2 nm s^−1^. The remainder solution in the 3 mL plastic vial (together with the carrier located at the bottom) and the solution in the cuvette were transferred back to the 10 mL of solution where the drug release experiment has been performed. This procedure was chosen for an efficient drug release process without changing the solution's volume and concentration and without losing the mass of drug carrier. The described procedure was performed at each selected time interval. After 4 min, the solution was irradiated with UV radiation with a wavelength of 254 nm to initiate the course of the photocleavage reaction with the subsequent release of the drug (see Figs. 1 and 2c). Before determining the concentration of NAP, the calibration curve was constructed based on Naproxen sodium solutions with different concentrations (see Fig. S1 in ESI). The linearity of the calibration curve was confirmed by the correlation coefficient *r*^*2*^ = 0.9999.

### In vivo release experiments

All the experiments performed on animals in this work were realized in adherence to ARRIVE guidelines and were approved by the National Veterinary and Food Administration of Slovak Republic and Animal Care Committee of P.J. Šafárik University in Košice, Slovak Republic (decision no. Ro-1064/19-221/3), according to the European Communities Council Directive 86/609/EEC and in compliance with current national legislation. Adult Wistar albino rat males of IGS strain (Charles River Laboratories) obtained from Velaz (Prague, Czech Republic) weigthing 400–500 g were used in the experiment. Animals were housed under standard laboratory conditions with a 12/12 h light/dark cycle and with ad libitum access to water and a standard laboratory diet for rats and mice (Altromin, Velaz, Prague, Czech Republic). Animals were *i.p.* injected with a single dose of Tiletamin and Zolazepam (dosage: 20 mg per kg of bodyweight, Zoletil, Virbac, Czech Republic) for analgesia and anaesthesia before administration of the experimental solution. After cessation of reflexes, tails of experimental animals were soaked in warm water for vasodilatation. First, to obtain the results on the Naproxen sodium salt bioavailability in blood serum of rats using HPLC, a group of animals (group a, n = 12) was injected by Naproxen sodium (10 mg/kg of bodyweight) dissolved in sterile saline and sacrificed by cervical decapitation after 1, 4, 8 and 24 h. In the next step, group of animals (group b, n = 12) was injected with sterile saline containing dispersed modified MCM-41 silica, which pores filled by NAP (in order to achieve the dosage of 10 mg/kg) were opened by irradiation of nanoparticles using UV light (*λ* = 254 nm) before preparation of the experimental solution (sample MCM-41-CA/NAP (open)) to analyze the release of Naproxen sodium salt from silica during the same period of time in in vivo conditions. To analyze the pore-closure stability of modified MCM-1 silica in in vivo conditions, a group of animals (group c, n = 12) was injected with sterile saline containing dispersed modified MCM-41 nanoparticles, which pores filled by NAP (10 mg/kg of bodyweight) were closed by irradiation of nanoparticles using UV light (*λ* = 365 nm) before preparation of the experimental solution (sample MCM-41-CA/NAP (closed)), surviving 1, 4, 8 and 24 h. Finally, to analyze the effectivity of pore-opening in vivo using UV light, a group of animals (group d, n = 6) was injected with sterile saline containing dispersed modified MCM-41 nanoparticles, which pores filled by Naproxen sodium (10 mg/kg of bodyweight) were closed using irradiation of nanoparticles by UV light (*λ* = 365 nm) before preparation of the experimental solution (sample MCM-41-CA/NAP (closed)). After 1 or 24 h in circulation, *vena saphena magna* of the animal hindlimb was irradiated (*λ* = 254 nm).

All substances used in the experiment were dissolved/dispersed in 200 µl of sterile saline. After *i.v.* injection of the experimental solution into the tail vein, another 300 µl of sterile saline was inspired into the insulin syringe and injected into the tail vein of animals to secure the administration of all the components present in the syringe. For irradiation of animals using UV light (group d) the fur on hindlimbs of experimental animals was shaved to expose both saphenous veins. The eyes of experimental animals were covered by eye ointment (Corneregel, Bausch Health, Laval, Canada) to avoid the drying of cornea and the head of animals was inserted into a prepared protection roll. Whole animals under anaesthesia were transferred into a black box equipped with a UV lamp (UV lamp E-580 (40 W) operated at 254 and 365 nm) and irradiated by UV light (*λ* = 254 nm) for 30 min to analyze the effectivity of pore opening in modified nanoparticles. Animals were sacrificed by cervical decapitation in 1 h after irradiation of animals using UV light (group d). In each experimental group, 3 animals were used (n = 3) per time point. After cervical decapitation of anesthetised animals, arteriovenous blood was collected, left to coagulates and centrifuged at 1200 relative centrifugal force (RCF) for 20 min. Blood serum, red blood cells with buffy coat (blood clot) and liver were collected and stored in the freezer at − 20 °C until further processing.

### Solid-phase extraction procedure

Naproxen sodium salt was preconcentrated and isolated from rat blood serum by solid-phase extraction (SPE) and analysed by HPLC. For SPE, the vacuum manifold purchased from Machery-Nagel (SPE manifold CHROMABOND®, MACHEREY–NAGEL, Dueren, Germany) was connected to a vacuum pump. 1 mL of the blood serum sample or calibration standards was diluted with 3 mL of 100 mM CH_3_COOH (99.8%, Acros Organics, CAS: 64-19-7, CN: 148930010). The mixture was vortexed for 1 min C18 SPE columns (Strata C18, 500 mg/6 mL) were initially activated using 3 mL of methanol (HPLC Plus, ≥ 99.9%, Sigma-Aldrich, CAS: 67-56-1, CN: 646377), and then conditioned using 3 mL of water and 1 mL of 100 mM CH_3_COOH. After washing and conditioning steps, the serum sample passed through the SPE columns at a speed of 1 mL/min. The columns were prewashed with 6 mL of a solution containing 100 mM CH_3_COOH and water (50:50, *v/v*) and dried by applying vacuum for 15 min (10-inch Hg). Next, the extracted sample was eluted with 3 mL of a solution of ethyl acetate (HPLC Plus, ≥ 99.9%, Sigma-Aldrich, CAS: 141-78-6, CN: 650528) and methanol (90:10, *v/v*) with a flow rate of 1 mL/min. The extract was then evaporated to dryness under a stream of nitrogen and the sample residue was dissolved in 1 mL of acetonitrile (HPLC Plus, ≥ 99.9%, Sigma-Aldrich, CAS: 75-05-8, CN: 34998). Directly, 1 μL of the Naproxen sodium extracts or standard solutions was injected into the HPLC system.

### HPLC conditions

The HPLC separation was performed on a reversed-phase C18 Kinetex column, particle size 2.6 μm, 150 × 4.6 mm protected by a Security Guard column (Phenomenex, Torrance, CA, USA). The flow rate was 1.0 mL/min while using isocratic elution with the mobile phase acetonitrile—phosphate buffer (PBS, Acros Organics, CAS: 7447-40-7, CN: BP399-1, *pH* = 3.0) 55:45, v/v mixture and degassed before use. The mobile phase was degassed in an ultrasonic water bath for 5 min. The evaporated residue was dissolved in 1 mL of acetonitrile and 1 μL of the solution was injected into the HPLC instrument for analysis. Before injection into HPLC, a 25 mm diameter sterile syringe filter with a 0.22 μm pore size hydrophilic PVDF membrane was used for filtration of dissolved solution. The fluorescence detection was performed at an excitation wavelength of 270 nm and an emission wavelength of 340 nm. All analyses were performed at laboratory temperature (25 °C). Obtained chromatograms of Naproxen sodium detected in rat blood serum for pure drug and prepared carriers are presented in Fig. S2 in ESI.

### Characterization

Infrared spectra of prepared and calcined biological materials were measured by FT-IR spectroscopy on Nicolet 6700 from Thermo Scientific (Thermo Fisher Scientific, Waltham, MA, USA) using the ATR technique in the wavelength range of 4000–400 cm^−1^. All spectra were recorded with a resolution of 4 cm^−1^ by collecting 64 scans for a single spectrum at ambient temperature. The obtained IR data were analysed using OMNIC, Version 8.2.0.387 software (Thermo Fisher Scientific, Waltham, MA, USA)^31^.

The thermal properties of prepared materials were determined by thermal analysis (TG) with TGA Q500 from TA Instruments (TA Instruments, ew Castle, DE, USA) with a heating rate of 10 °C min^−1^ and in the temperature range from ambient temperature to 800 °C in an air atmosphere with a flow rate of 50 cm^3^ min^−1^ using platinum crucibles. All TG curves were normalized to 100 °C to remove weight loss corresponding to the solvent molecules, which was subsequently redistributed to the mass change of organic molecules and the residual mass^28^. The obtained TG data were analysed using OriginPro 8, Version 8.0891 software (OriginLab Corporation, Northampton, MA, USA)^32^.

Scanning transmission electron microscopy (STEM) and energy dispersive X-ray (EDX) images were taken by JEOL TEM 2100F UHR microscope operated at 200 kV. The microscope was run in STEM-BF mode. All samples for TEM observations were deposited on copper support grid covered with holey carbon film. The X-ray signal generated by samples was processed using INCA Suite software, version 4.15 (Oxford Instruments, High Wycombe, United Kingdom)^33^. Structures of samples were observed in STEM bright field mode employing JEOL Simple Image Viewer software, version 1.3.4 (JEOL, Tokio, Japan)^34^.

Adsorption/desorption isotherms measured at − 196 °C were performed on ASAP 2020 Micromeritics apparatus (Micromeritics, Norcross, GA, USA). Before nitrogen adsorption/desorption experiments, samples were outgassed at different temperatures (150 °C for MCM-41 and 60 °C for surface modified and drug-loaded materials). The BET specific surface area (*S*_*BET*_) of each sample was evaluated using adsorption data in a *p*/*p*_0_ range from 0.05 to 0.20. Pore volume (*V*_*p*_) and pore size diameter (*d*) were calculated using the BJH (Barrett-Joyner-Halenda) method from the desorption branch of the corresponding nitrogen isotherm. The obtained adsorption data were analysed using ASAP 2020 Plus, Version 2.00 software (Micromeritics, Norcross, GA, USA)^35^.

The amount of Naproxen sodium salt released during the in vitro experiments and the reversible photo-dimerization of CA ligand were determined by UV–VIS spectroscopy on a Specord 250 UV–VIS spectrometer from Analytik Jena AG (Analytik Jena GmbH, Jena, Germany) in a wavelength range of 200–500 nm. The obtained UV–VIS data were analysed using ASpect UV, Version 1.4 software (Analytik Jena GmbH, Jena, Germany)^36^.

UV lamp KRÜSS E-580 (40W, A.KRÜSS Optronic GmbH, Hamburg, Germany) was used as a source of UV radiation at 254 and 365 nm for in vitro and in vivo experiments.

High-pressure liquid chromatography (HPLC) was used to determine the concentration of drug in the extracted biological samples. The HPLC Dionex UltiMate 3000 RS system from Thermo Fisher Scientific (Thermo Fisher Scientific, Waltham, MA, USA) consisted of a quaternary pump, degasser, automated injector, column oven, FLD detector and obtained data were analysed using Chromeleon Chromatography Data System, Version 7.2 software (Thermo Fisher Scientific, Waltham, MA, USA). All graphs in the manuscript were drawn using OriginPro 8, Version 8.0891 software (OriginLab Corporation, Northampton, MA, USA)^32^.

### Statistical analysis

The data were analyzed by One-way analysis of variance (ANOVA) followed by Tukey–Kramer post-hoc tests for multiple comparisons. Differences between individual studied groups of samples in certain time points were statistically significant at *P* < 0.05 (*), *P* < 0.01 (**), *P* < 0.001 (***).

## Results and discussion

### Photosensitive DDS preparation and characterization

**Figure 3:**
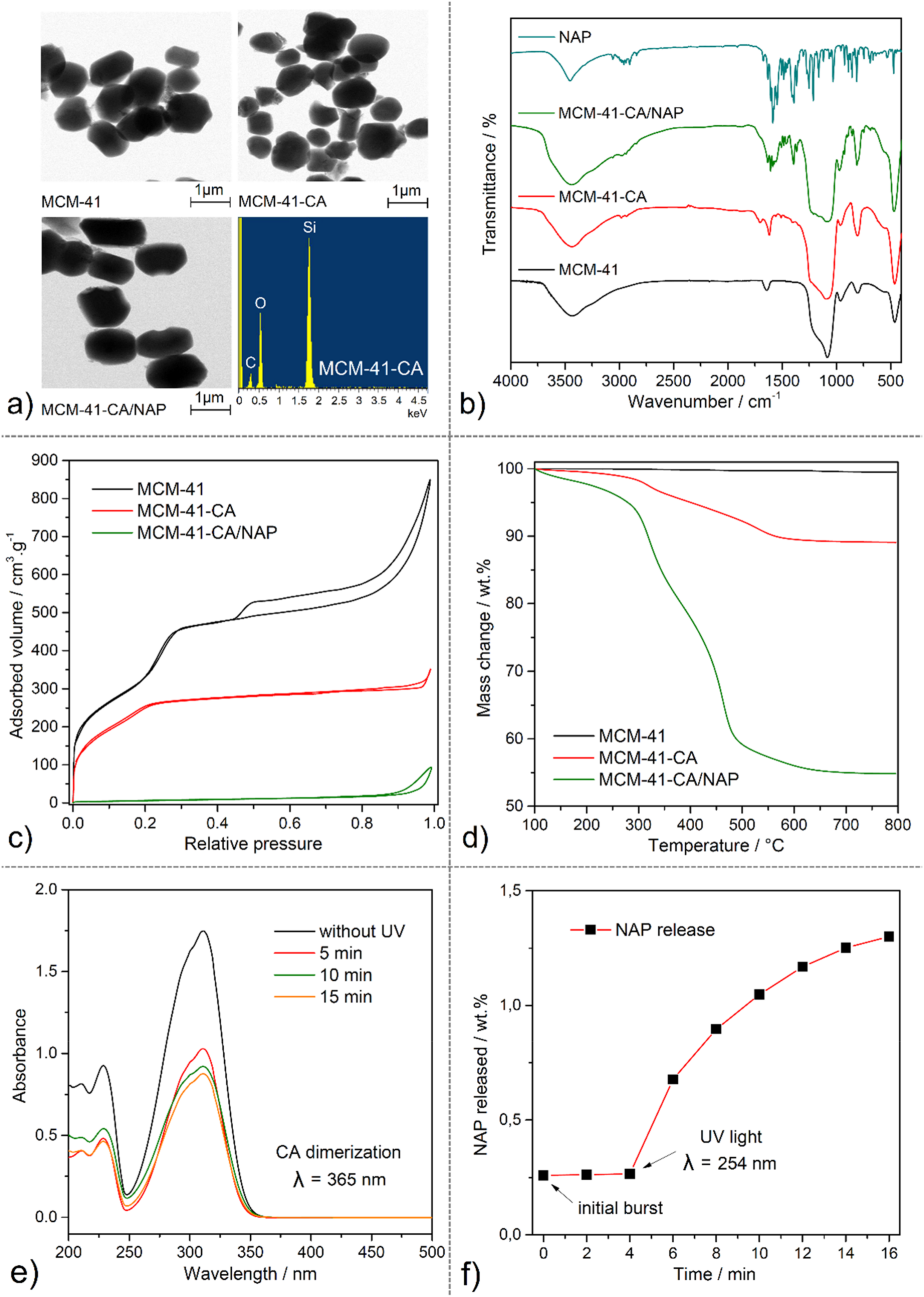
Characterization of MCM-41, MCM-41-CA and MCM-41-CA/NAP materials: (**a**) STEM and EDX images, (**b**) IR spectra, (**c**) nitrogen adsorption/desorption isotherms and (**d**) thermogravimetric curves. (**e**) UV spectra of CA´s photodimerization process (*λ* = 365 nm) and (f) in vitro release of NAP from MCM-41-CA/NAP (closed) before and after UV irradiation with *λ* = 254 nm.

The mesoporous MCM-41 material was prepared according to the procedure described in^30^ and the photosensitive ligand CA according to^27^. The CA molecules were attached to the surface of MCM-41 material by the grafting process (MCM-41-CA). The drug, Naproxen sodium salt, was encapsulated by the impregnation method into mesoporous silica matrix (MCM-41-CA/NAP). The prepared materials were characterized by several analytical techniques, the results of which are given below and presented in Fig. 3.

From scanning transmission electron microscopy (STEM) of MCM-41, MCM-41-CA and MCM-41-CA/NAP, it is evident that the size of the prepared carrier´s particles is approximately 1 μm in length and the particles have a rod-like shape (see Fig. 3a). Moreover, the grafting process and drug encapsulation did not affect particle morphology and their size. EDX record of MCM-41-CA/NAP material also confirmed the presence of the required elements, silicon, oxygen and carbon (see Fig. 3a).

The successful synthesis and calcination process of MCM-41, functionalization with CA groups in the case of MCM-41-CA and subsequently NAP loading were evidenced by IR spectroscopy (see Fig. 3b). The IR spectra of all prepared materials showed the characteristic silica vibrations at 1083 and 802 cm^−1^ corresponding to Si–O–Si antisymmetric (*ν*_*as*_(SiOSi)) and symmetric (*ν*_*s*_(SiOSi)) stretching vibrations, respectively and bending (*δ*(SiOSi)) vibration at 470 cm^−1^. Grafted CA molecules in the MCM-41-CA sample reflects the presence of aromatic stretching C–H vibration (*ν*(CH)_ar_) at 3015 cm^−1^, several aliphatic stretching C–H vibrations (*ν*(CH)_aliph_) in the range of 3000–2800 cm^−1^ and carbonyl stretching vibration (*ν*(C=O)) at 1708 cm^−1^. Figure 3b also shows a comparison of the IR spectra of Naproxen sodium salt and MCM-41-CA/NAP, which confirm the presence of NAP molecules in the pores of MCM-41-CA. The presence of NAP is evident from the antisymmetric (*ν*_*as*_(COO^−^)) and symmetric (*ν*_*s*_(COO^−^)) stretching vibration of carboxylate group at 1633 and 1392 cm^−1^ and stretching aromatic C=C absorption bands (*ν*(C=C)_ar_) at 1601, 1582 and 1504 cm^−1^.

Nitrogen adsorption experiments were performed to evaluate the textural properties (specific surface area (*S*_*BET*_), pore volume (*V*_*p*_) and pore diameter (d) see Table 1) of MCM-41 and their changes upon grafting process with CA molecules and drug loading (see Fig. 3c). Figure 3c shows type *IV* isotherm for MCM-41 with a surface area of 923 m^2^ g^−1^ and pore volume 0.95 cm^3^ g^−1^. The adsorption isotherm for MCM-41-CA shows a significant reduction of surface area (638 m^2^ g^−1^) and pore volume (0.52 cm^3^ g^−1^) but the same type *IV* feature as the original calcined MCM-41 material, indicating that upon post-synthetic modification the ordered structure of MCM-41 remains unchanged. In contrast to this, a significant decrease in surface area (19 m^2^ g^−1^) for MCM-41-CA/NAP was observed, suggesting that the mesoporous structure of MCM-41 was completely blocked upon drug loading.

**Table 1:** Calculated textural parameters (specific surface area (*S*_*BET*_), pore volume (*V*_*p*_) and pore diameter (d)) of prepared materials from N_2_ adsorption/desorption isotherms measured at 77 K.

Sample	Surface area	Pore volume	Pore size
	*S*_*BET*_	*V*_*p*_	*d*
	m^2^ g^−1^	cm^3^ g^−1^	nm
MCM-41	923	0.95	2.52
MCM-41-CA	638	0.52	2.35
MCM-41-NAP	19	0.04	–

The amount of grafted CA molecules and the encapsulated drug was quantified by thermogravimetric analysis (TGA, see Fig. 3d). The TG curve of MCM-41 indicates successful removal of surfactant molecules in as-synthesized MCM-41 material after calcination processes as no significant weight loss in the whole temperature range was observed. On the TG curve of MCM-41-CA material, a weight loss of 10.12 wt% was detected, which corresponds to the binding of 101.2 mg g^−1^ of CA molecules on the surface of the support. The amount of encapsulated drug was determined from the difference between the weight loss of MCM-41-CA and the drug-containing sample, MCM-41-CA/NAP. According to the obtained TG data, the loading capacity of MCM-41-CA was calculated, which is defined as the amount of drug-loaded per unit weight of the carrier. A total weight loss of 45.14 wt% was observed on the TG curve of MCM-41-CA/NAP, which corresponds to the loading capacity of 350.2 mg of NAP per 1 g of the drug-loaded carrier (35.02 wt%). The stated calculated loading capacity was used as 100% in both, in vitro and in vivo drug release experiments. The entrapment efficiency (EE) of 73.0 wt.% was calculated as the difference between the initial drug quantity (600 mg of NAP) and the quantity of drug in the supernatant after the encapsulation process with respect to the total quantity of NAP incorporated in the carrier (161.7 mg of NAP per 300 mg of MCM-41-CA).

The photo-dimerization reaction of CA molecules was investigated by UV spectroscopy. The UV spectrum of CA contains three intensive absorption bands with maxima at 211, 229 and 312 nm, which correspond to the π-π* and n-π* electron transitions (see Fig. 3e). After irradiation of CA molecules with UV light (365 nm), the intensity of mentioned absorption bands decreased with increasing irradiation time. During the photochemical reaction, a [2π + 2π] cyclization reaction occurs between ethylene double bonds of two CA molecules to form a cyclobutane ring, as shown in Figs. 1, 2c. As a result of the photocyclization, the reaction is a disruption in the conjugation π-electron system, which is reflexed by a decrease in the absorbance of all the electron transitions. Observed results are in good agreement with previously published data^27^.

In vitro drug release experiment (see Fig. 3f) was performed in saline solution at *pH* = 7.4 representing simulated body fluid. The drug released amounts at various time intervals without and using UV radiation (254 nm) was analysed by UV spectroscopy. The calculated amounts of NAP were determined using a calibration curve (see Fig. S1 in ESI). As can be seen from Fig. 3f, in the first two minutes of the in vitro experiment, 13 wt.% of NAP, the so-called "burst release" of the drug from the surface of MCM-41-CA/NAP (closed) was observed. In the next six minutes of the experiment, there is no significant change in the release profile of NAP. After irradiation of the suspension with UV light with a wavelength of 254 nm, the pores of the material are opened and the drug is subsequently released. Up to 10 min after irradiation of the material, 68 wt. % of Naproxen sodium salt was released. Prepared DDS showed the desired effect of an external stimulus represented by UV radiation and the drug release. Therefore, the behaviour and the effectivity of the MCM-41-CA/NAP system were subsequently studied by in vivo experiments.

### In vivo experiments

In our recent works^22,23,28,29^ we have studied in vitro biocompatibility and cytotoxicity of various mesoporous silica materials, non-modified or surface ligand modified. We did cytotoxicity experiments on the U87 MG and SKBR3 cancer cells using the microscopic techniques, flow-cytometry, MTT assay, apoptosis assay and CAM assay. The results showed that cytotoxicity of anticancer drug 5-fluorouracil (5-FU) towards cancer cells can be increased by loading of 5-FU into the mesoporous silica. Moreover, the cytotoxicity of 5-FU against cancer cells was higher when 5-FU was delivered in silica in co-adsorption with naproxen (NAP), compared to experiments when 5-FU was delivered alone^23^. Thus, it was shown, that designed silica-based systems work safely as a cargo for anticancer drugs. These results in vitro are encouraging; however, the toxicity of MSNs nanoparticles in vivo still must be carefully elucidated. There were several reviews published, discussing and showing the toxicity of silica particles in vivo, e.g.^37–39^. As for mesoporous silica (MSNs), Fu et al.^40^ studied the absorption, distribution, excretion and toxicity of mesoporous silica nanoparticles in mice following different exposure routes. They found that MSNs can be hardly absorbed in a short time after hypodermic and intramuscular administration. However, the pathological examinations illustrated that MSNs possessed good tissue biocompatibility after oral and intravenous injection and oral and intravenous administration were found to be safe routes for possible biomedical application of mesoporous silica nanoparticles^40^. Li et al. showed that absorption and toxicity of MSNs depends on their size and shape^41^. They studied three types of MSNs with different aspect ratios (1, 1.75 and 5) after oral administration. With the decrease of aspect ratio, the systematic absorption by small intestine and other organs increased and the urinary excretion decreased. Particle shape dependent renal toxicity of MSNs was observed^41^. Similar MSNs shape determined behaviour was described by other authors^42^. Chen et al. concluded in their study, that MSNs cause toxicity to immune cells and tissues. The main mechanisms were pro-inflammatory responses, oxidative stress, autophagy. However, the modifications of surface and shape may mitigate the toxicity effects^43^. Anyway, also opposite results have been reported about the toxicity MSNs. Bhavsar et al. studied in vitro and in vivo safety and degradation of MSNs^19^. They found that MSNs are safe after i.v. administration up to 40 mg/kg and cause no acute or chronic toxicities. Moreover, it was found that MSNs are degradable, and they are excreted from Wistar rats within 4 days after i.v. injection^19^. It is clear, that further studies are needed to fully understand the toxicity of porous silica particles (MSNs). However, the aim of the present study is not to study toxicity of silica carrier, but the functionality, efficiency, and photosensitivity of light driven gate opening/closing mechanism of MSNs DDS in living organisms.

**Figure 4:**
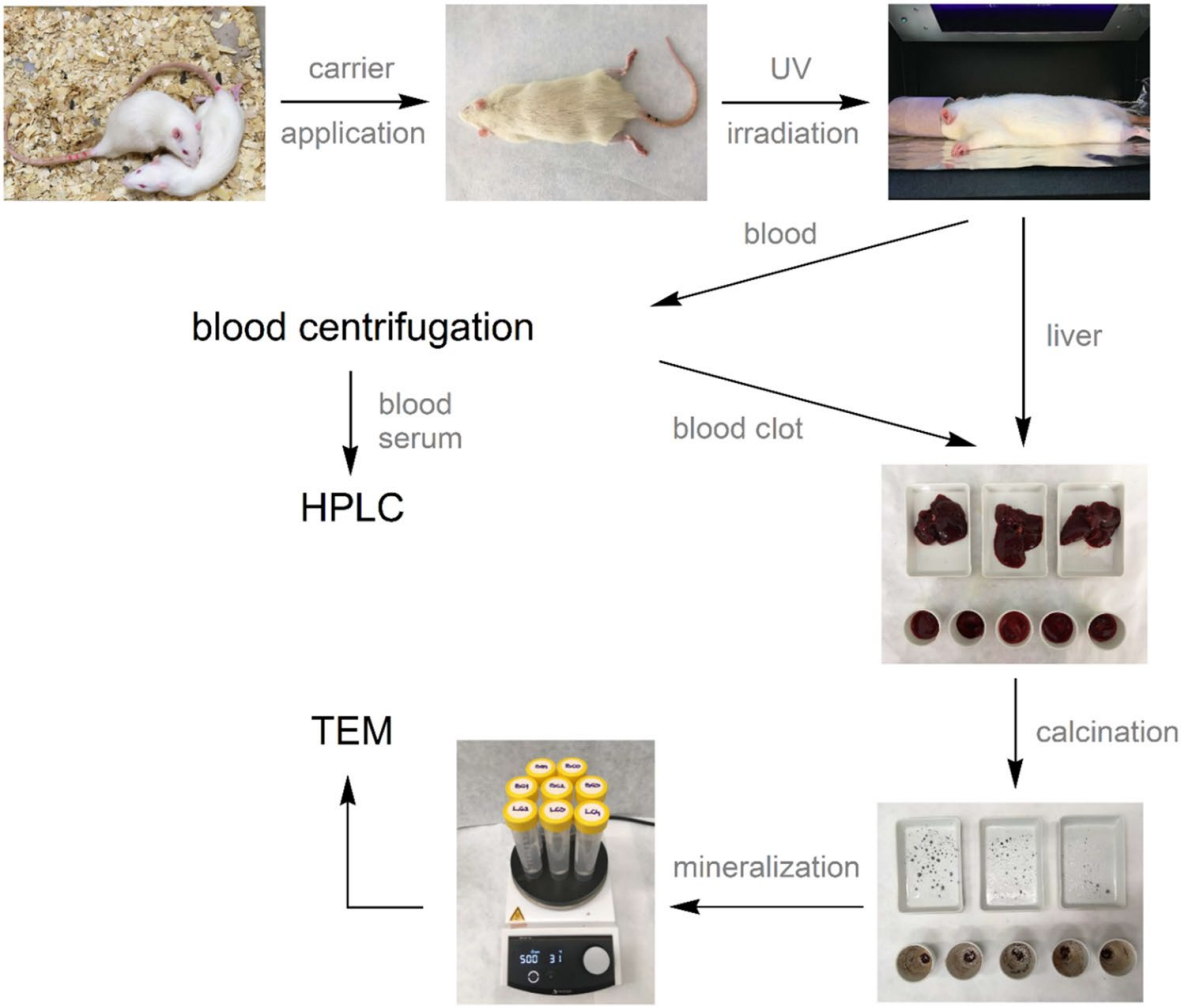
Scheme of in vivo experiments and quantitative/qualitative analysis during the study of effectivity of pore opening in vivo using UV light (for experiment on group of animals denoted as d.) in section “In vivo release experiments”).

Initially, to confirm the photosensitivity and efficiency of the prepared MCM-41-CA carrier in the living organism, represented by adult Wistar albino rats, in vivo experiments were performed. Experimental design of the in vivo experiments and the subsequent quantitative and qualitative analyses of the isolated biological materials are shown in Fig. 4. MCM-41-CA/NAP was initially administered to the rats and the animals survived at different time intervals (1, 4, 8 and 24 h after DDS administration). The rats were left either untreated or irradiated with UV light (*λ* = 254 nm) after administration of the carrier. Subsequently, blood was isolated from rats, from which blood serum and blood cells (blood clot) were separated by centrifugation. The concentration of Naproxen sodium salt was determined from the blood serum by HPLC. Moreover, livers and blood clots were also isolated and further calcined and mineralized. The samples thus prepared were analysed using FSEM and EDX by which the presence of carrier particles and their morphology were determined.

**Figure 5:**
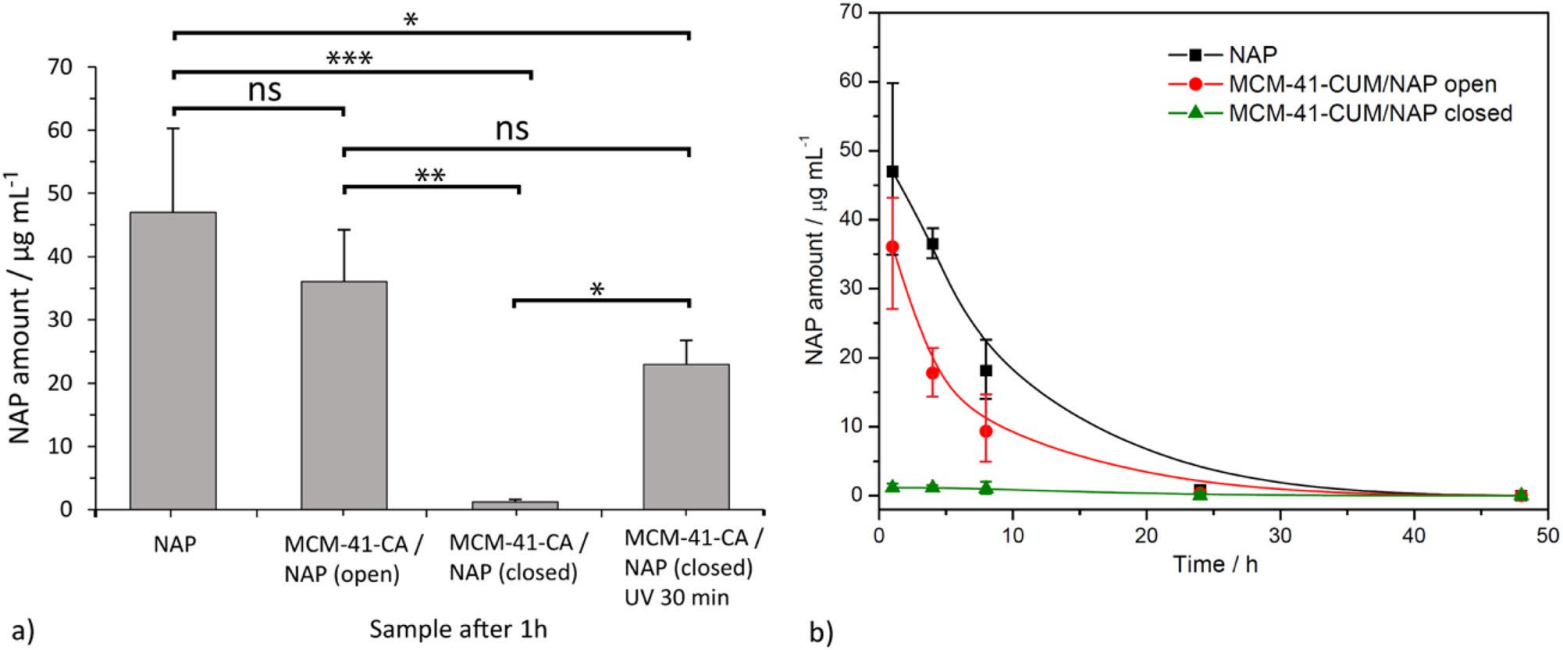
(**a**) The detected concentrations of Naproxen sodium salt in rat blood serum after one hour of survival for pure drug, MCM-41-CA/NAP with open and closed pores, and one hour survival after 30 min irradiation of the closed-form with UV light. (**b**) Detected concentrations of Naproxen sodium salt in blood serum of prepared materials / pure drug at different time intervals.

Measured concentration of the drug in the blood serum of rats, 1 h after drug/carrier administration shows Fig. 5a. As can be seen, the highest concentration of 46.97 ± 13.29 µg/mL was found for the pure drug. From the MCM-41-CA (open) carrier with CA molecules in the open conformation, 36.05 ± 8.17 µg/mL of NAP and from material MCM-41-CA (closed) with CA molecules in the closed conformation, 1.17 ± 0.39 µg/mL of NAP (“burst release”) was detected. The 22.96 ± 3.80 µg/mL of NAP was found for the sample MCM-41-CA/NAP in the closed conformation circulating in system for 1 h, irradiated by UV-light of a wavelength 254 nm for 30 min and subsequent survival of 1 h (photo-cleavage, opening the pores). The described experimental results confirmed that the photosensitive molecules CA grafted on the MCM-41 silica surface are effective even in in vivo environment.

Moreover, additional in vivo experiments were performed: after 24 h of MCM-41-CA/NAP (closed) administration, rats were irradiated with UV light. The HPLC results showed, that the concentration of Naproxen sodium salt was below the detection limit. We assume that after 24 h, the carrier’s particles are absorbed in the liver and the exposure to UV light does not reach the photoreaction of CA molecules, as UV radiation has a low penetration capacity. The described assumption was confirmed by TEM measurements of blood clots and isolated livers (see results below).

Based on the positive experimental results described above, the in vivo experiments were subsequently performed, in which pure drug and MCM-41-CA materials with open and closed pores were *i.v.* administered to rats. The concentration of the drug in the blood serum for all samples was analysed at selected time points (1, 4, 8 and 24 h). The dependence of the drug concentration in the blood serum as a function of time is shown in Fig. 5b. After the administration of the pure drug (46.97 ± 13.29 µg/mL NAP after 1 h), a gradual decrease of Naproxen sodium salt in blood serum was due to its metabolic conversion. After 8 h, the amount of Naproxen sodium was 18.14 ± 3.36 µg/mL, indicating a half-life of *i.v.* administered NAP is shorter than 8 h. Finally, only a minimal amount of drug was detected after 24 h, 0.85 ± 0.50 µg/mL NAP. A lower amount of Naproxen sodium (36.05 ± 8.17 µg/mL) was obtained from MCM-41-CA/NAP (open) 1 h after the *i.v.* administration, which, similarly to the pure drug, decreased with increasing time up to 24 h (0.39 ± 0.10 µg/mL NAP). In the case of closed-pore material, the only minimal release of NAP (1.17 ± 0.39 µg/mL after 1 h) was initially observed due to the effective pore closure by CA molecules.

As it can be seen from the experiment the half-life of NAP was about 7–8 h after *i.v.* administration of dose 10 mg/kg of bodyweight. This period well agrees with the published results of NAP excretion from rats, which half-life in plasma after administration of 3 mg/kg was estimated to 5.1 ± 1.8 h^44^ or 5.31 ± 0.9 h after *i.v.* administration of 6 mg/kg^45^. The metabolism of Naproxen sodium salt is simple: it is excreted almost entirely in the urine as the native molecule, its oxidative 6-desmethyl metabolite and their respective conjugates^46^. The mean half-life of the drug in man was estimated to 12–15 h. The pharmacokinetics of NAP in animals differ substantially from human, since , the half-life in dogs was estimated to 35–74 h, 4–8 h in horses and 5.31 h in rats^44,45,47^.

**Figure 6:**
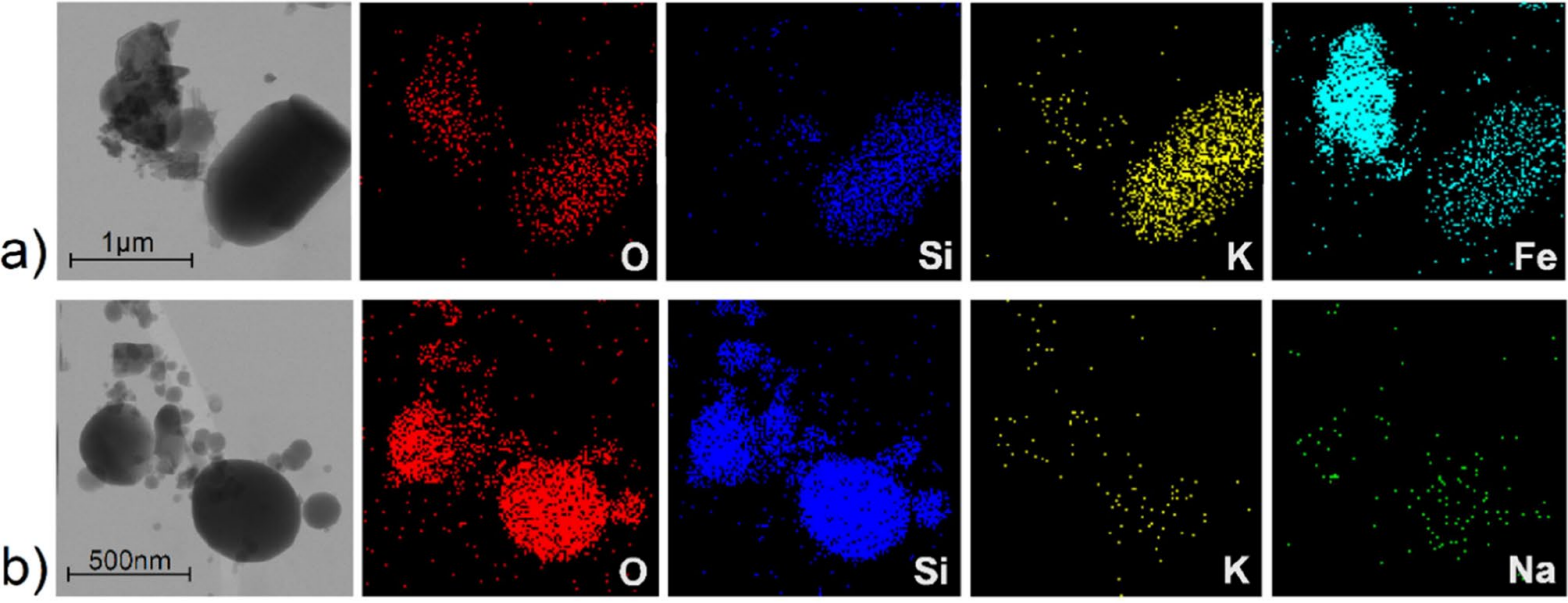
STEM and EDX images of isolated materials from (**a**) blood clot after 1 h and (**b**) liver after 8 h of survival.

In addition to determining the amount of drug in the blood serum of rats, the blood clots and livers were analysed to study the presence/localization and morphology changes of silica particles. Mentioned samples were isolated from rats after 1 and 8 h of survival. The biological materials were initially calcined by slow heating to 600 °C to remove organic components and then mineralized by stirring in HCl at *pH* = 2 to remove soluble inorganic compounds. Isolated materials were studied by STEM, EDX and IR and obtained results are shown in Fig. 6 and Fig. S3. In the isolated materials after 1 h, no silica particles were present in the liver, while they were detected in the blood (see the comparison of IR spectra in Fig. S3 in ESI of MCM-41 and calcined biological materials collected after 1 h of survival).

As can be seen from Fig. 6a, in the blood circulatory system of rats, the particle size was maintained and no morphological changes were observed. The presence of SiO_2_ particles was proved by elemental analysis using EDX, which confirmed the presence of oxygen and silicon (see red and blue colours in Fig. 6a). In addition, the presence of potassium (see yellow colour in Fig. 6a), which is part of the blood plasma and iron (see cyan colour in Fig. 6a) from red blood cells, has been observed. Iron was present in the isolated material in the form of Fe_2_O_3_, which forms separate isolated particles or is found in the pores of the MCM-41 material (see cyan colour in Fig. 6a). In the isolated materials after 8 h of survival, the carrier particles were found in the liver (Fig. 6b), but not in the blood (not shown). The gradual hydrolysis of the particles was observed, which resulted in their size reduction from the original 1 μm diameter to approximately 500 nm and smaller. Morphological changes also occurred as the particles changed from the original rod-shaped form to spherical (Fig. 6a,b). We identified that particles are subsequently cleared from blood stream and particles, which are present in the liver undergo gradual degradation (Fig. 6). Since the nanoparticles were partially degraded, we may assume that the nanoparticles do not accumulate in the liver, but are gradually degraded over a time. This result is in accordance with other studies dealing with safety, toxicity and degradation of MSNs, showing dissolution based degradation of MSNs nanoparticles^19^.

## Conclusions

The present study examined the effectivity of designed photo-sensitive DDS in in vivo environment, after its administration into the blood circulation of rats. DDS was formed by the mesoporous material MCM-41, which was surface-modified with a cinnamic acid derivative and loaded with Naproxen sodium salt. The CA molecules located on the surface of MCM-41 served as gatekeepers through which the drug is blocked/released by UV irradiation. CA molecules undergo a photo-dimerization reaction by radiation with a wavelength higher than 365 nm, through which the drug molecules were encapsulated in the pores. Exposure to UV at a lower wavelength (254 nm) results in a photo-cleavage reaction, which opens the pores of the material and caused the drug release. The prepared composite material was characterized by several analytical techniques: IR confirmed the presence of the desired components, textural properties were studied by nitrogen adsorption/desorption measurements, morphology and elemental analysis of the prepared particles were analysed by STEM and EDX. The amount of grafted CA molecules and the encapsulated drug was determined by TG analysis. The mechanism of photo-dimerization and photo-cleavage of CA molecules was confirmed by UV measurements as well as in vitro drug release by exposure to UV light. The photosensitivity of CA molecules was also successfully confirmed in in vivo experiments, which demonstrated the drug release in the blood serum by the action of UV light. Initially, the metabolic degradation of Naproxen sodium salt was studied by administering the pure drug and subsequently the drug stored in MSNs carriers with open and closed pores. It was confirmed that there was a gradual drug release from the open pore carrier (MCM-41-CA/NAP (open)) and the closed pore material (MCM-41-CA/NAP (closed)) showed minimal drug release, only due to the initial burst effect. Subsequently, UV irradiation experiments were performed in rats after application of MCM-41-CA/NAP (closed) to confirm the photochemical reaction in the living organism, which was reflected by the release of more NAP. The localization of particles in the body of rats (blood, liver) and the study of change in their morphology after 1 h and 8 h after the application of the carriers were investigated by STEM and EDX. Measurements showed that after 1 h the particles were present in the blood and after 8 h in the liver. No morphological changes in the original rod-like shape of the particles in the blood after 1 h were observed. However, after 8 h the particles of the MSNs carrier were present in the liver, while shrinking and changing to a spherical shape was observed, confirming the gradual degradation of MCM-41 material. According to the results described above, it can be concluded that the prepared photo-responsive DDS system is effective in the circulatory system of mammals. The present study is a pilot project that can serve as a study for further investigation and application of photosensitive DDSs in living systems.

## Supplementary Information

Supplementary Figures.
