# Supplementary material for: In vivo study of light-driven naproxen release from gated mesoporous silica drug delivery system
Source: Sci Rep. 2021 Oct 12;11:20191. doi: 10.1038/s41598-021-99678-y (PMC8511123; doi:10.1038/s41598-021-99678-y)
Supplement: Supplementary file 1 — Supplementary Figures. [file 41598_2021_99678_MOESM1_ESM.pdf]

Electronic Supplementary Information
(ESI)

In Vivo study of light-driven naproxen release from gated mesoporous silica drug delivery system

Miroslav Almáši[1], Anna Alexovič Matiašová[2], Monika Šuleková[3], Eva Beňová[1], Juraj Ševc[2], Lucia Váhovská[3], Maksym Lisnichuk[4], Vladimír Girman[4], Adriana Zeleňáková[4], Alexander Hudák[3], Vladimír Zeleňák[1]*

[1] Department of Inorganic Chemistry, Institute of Chemistry, Faculty of Science, P.J. Šafárik University, Moyzesova 11, SK-041 54 Košice, Slovakia
[2] Department of Cell Biology, Institute of Biology and Ecology, Faculty of Science, P. J. Šafárik University, Šrobárová 2, SK-041 80 Košice, Slovakia
[3] Department of Chemistry, Biochemistry and Biophysics, Institute of Pharmaceutical Chemistry, The University of Veterinary Medicine and Pharmacy, Komenského 73, SK-041 81 Košice, Slovakia
[4] Department of Condensed Matter Physics, Institute of Physics, Faculty of Science, P.J. Šafárik University, Park Angelinum 9, SK-041 54 Košice, Slovakia
*e-mail: vladimir.zelenak@upjs.sk

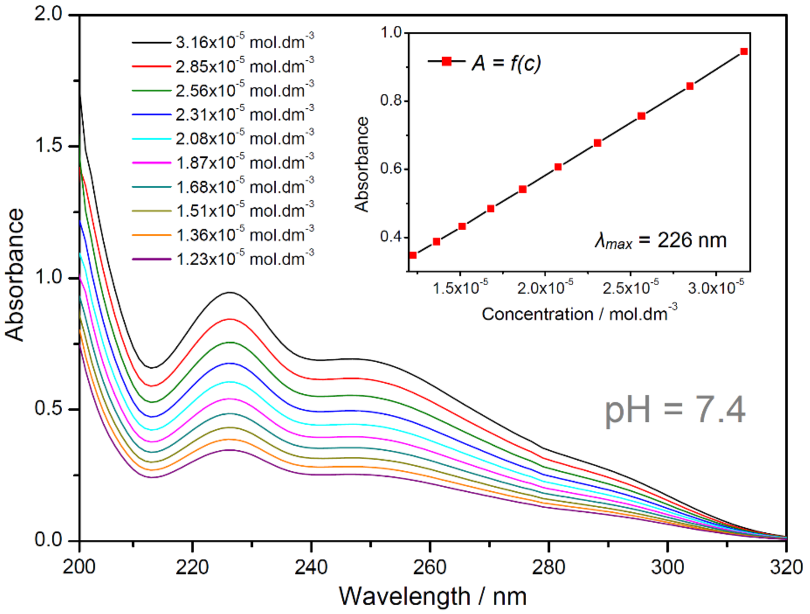

Fig. S1 UV spectra of naproxen sodium measured at pH = 7.4 at different concentrations. Inset shows a linear calibration curve A = *f*(c) with the corresponding λ_{max}.

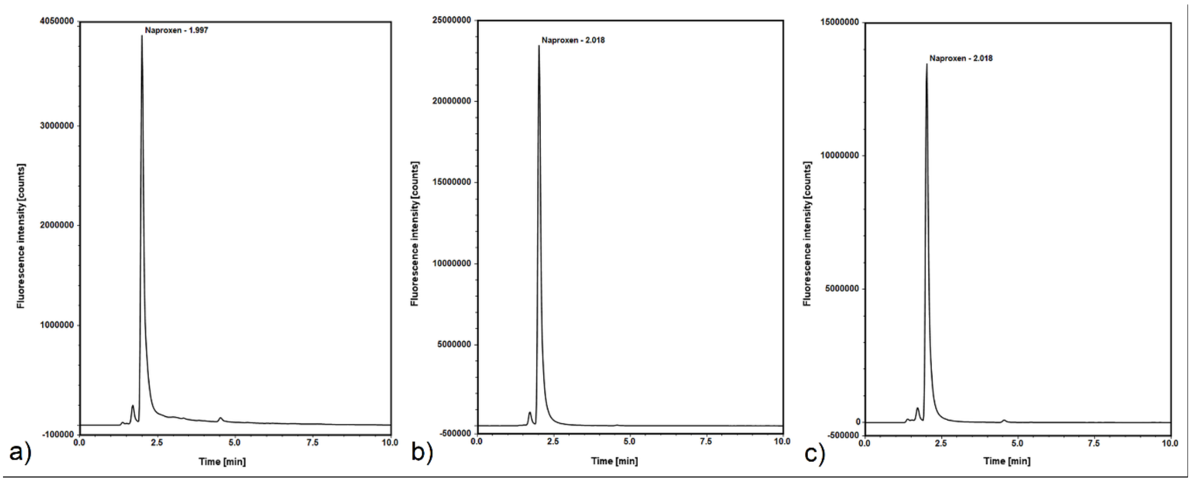

Fig. S2 Chromatograms of naproxen sodium detected in rat blood serum for a) pure drug, b) MCM-41-CA/NAP (open) and c) MCM-41-CA/NAP (closed).

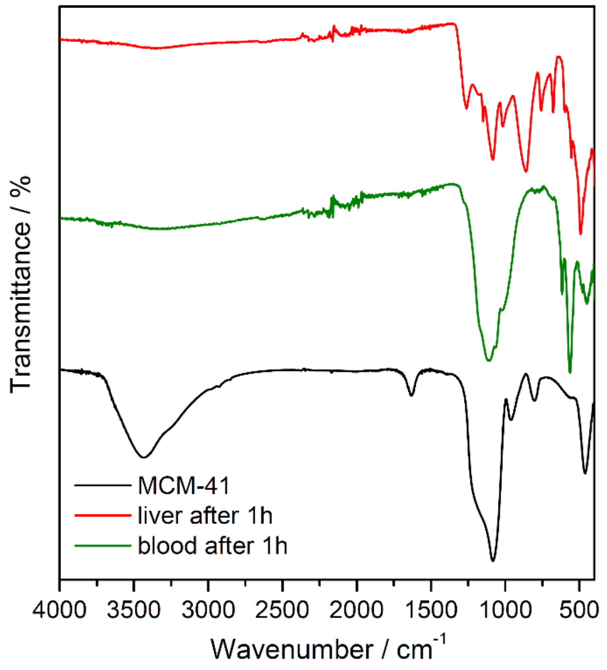

Fig S3. Comparison of the IR spectra of MCM-41 (black line), blood (green line) and liver (red line) obtained after 1 hour of survival and subsequent calcination.
